# Post-treatment periapical periodontitis X-ray versus CBCT - a case report

**Published:** 2016

**Authors:** P Perlea, C Nistor, I Suciu

**Affiliations:** * Department of Endodontology, “Carol Davila” University of Medicine and Pharmacy, Bucharest, Romania

**Keywords:** post treatment periodontitis, radiograph, CBCT

## Abstract

Post treatment periapical periodontitis is usually caused by residual microbes, due to poorly treated root canals and microleakage. Our clinical case proved that orthograde, single-visit endodontic re-treatment is the first choice for the clinician. Cone Beam Computed Tomography (CBCT) images detect the presence and the real extension of the periapical periodontitis and the outcome of the endodontic treatment, in terms of healing the bone defect. Compared with the limited 2-D data obtained by using the radiograph, the CBCT shows a 3-D image of the tooth, the root canal, and the surrounding tissue.

## Introduction

Persistent microbial infection within the endodontic system causes post-treatment apical periodontitis. Other factors involved in the persistent periapical radiolucency after root canal treatment are the extraradicular infection, extruded exogenous materials (filling materials, paper points, separated instruments) and true cysts with cholesterol crystals. A particular case is the scar tissue healing of the lesion, which is not considered a failure. The most frequent cause of persisting post treatment apical periodontitis is residual microbes in the apical region in both poorly and properly treated cases. The necrotic pulp offers the substrate for the microorganism, which is present as planktonic cells floating in the environment or organizing themselves in biofilms attached to the moist surface, in aggregates and co-aggregates [**1**,**2**,**3**]. Marginal leakage may also lead to the reinfection of the endodontic system [**4**]. The aim of the conservative retreatment is to remove the defective restorations and to eliminate the infection in the endodontic system.

## Aim

Our clinical case showed that the nonsurgical correct root canal retreatment could solve large apical periodontitis, which were previously considered cysts and were referred for endodontic surgery. The comparison between conventional periapical radiographic findings and CBCT images proved that CBCT provides more accurate tridimensional information regarding apical periodontitis.

## Clinical Case

Chief Complaint: A 48-year-old patient experienced an episode of pain associated with tooth 12, which was previously endodontically treated. The patient presented to his dentists who prescribed antibiotics for relief of the acute symptoms and then referred him to the endodontist. After the remission of the acute faze, the patient presented to the specialist for the management of the root canal treatment.

Medical History: unremarkable

Dental history: the dental treatment was extensive and of various quality

Clinical examination: Intraoral examination revealed a medium oral hygiene status. On the clinical examination, no draining sinus was observed on the buccal or palatal mucosa, which had a normal appearance. The periodontal probing depths were normal. A cast post was cemented without the proper root canal treatment. It was carried out 5 years earlier. The crown showed poor marginal adjustment. The tooth was not tender to percussion or buccal palpation or mobile.

**Fig. 1:**
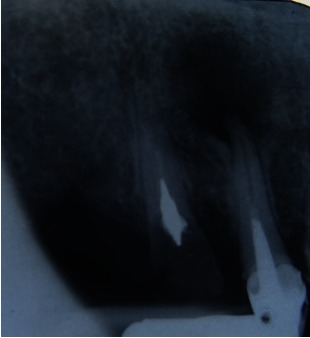
**Fig. 1**12 preoperative radiograph

**Fig. 2:**
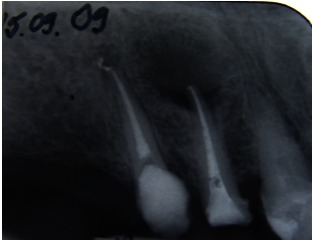
**Fig. 2**12 postoperative radiograph

The preoperative radiograph revealed the metal crown, a metal cast post, the root filling of the radiographic apex, which was short and a large periapical lesion (**Fig. 1,2**).

The diagnosis was chronic periapical periodontitis, associated with an existing root canal treatment. The size of the radiolucency suggested that the infection had been present for a considerable time.

The treatment options were root canal retreatment, surgical endodontics, or extraction. The patient was informed about the advantages and disadvantages of each approach and the risks for each procedure and chose to retain the tooth with conservative endodontic retreatment.

The first stage of the treatment involved the coronal disassembly, the crown was sectioned and removed, and consecutive the post was extricated after loosening with ultrasonic vibration. The inspection revealed that the remaining tooth structure was restorable.

The second stage of the treatment was root canal retreatment performed over one single appointment. Under local anesthesia, the tooth was isolated with rubber dam. Working with the operating microscope, the cemented post was removed, and it could then be identified that the obturation was performed with only the sealer, without the gutta percha. The canal was negotiated with small sized stainless steel hand files to the foramen. The working length was determined with the electronic apex locator, Morita, Tri Auto ZX, Japan, and confirmed on radiograph. The canal was shaped with ProTaper Universal, Dentsply, Maillefer, Switzerland and cleaned with heated NaoCl 5,25% and EDTA 17% (ethylenediamine tetra-acetic acid). Then the canal was dried with sterile paper points and obturated with AhPlus sealer, Dentsply, and warm vertical condensation of the gutta percha, continuous wave, Buchanan technique. A post space was prepared and a new crown installed, then the patient was monitored over time to assess the healing response. Over several years, follow-up radiographs showed a gradual resolution of the original periapical lesion. The best evolution occurred after a period between 12 and 18 months.

**Fig. 3:**
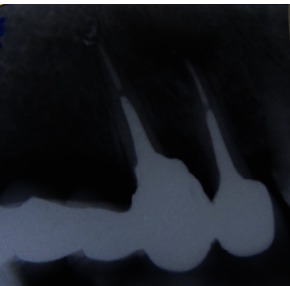
**Fig. 3**One year follow up

**Fig. 4:**
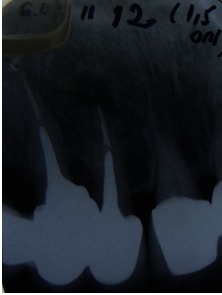
**Fig. 4**18 months follow up

**Fig. 5:**
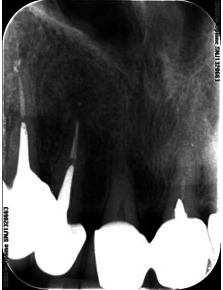
**Fig. 5**5 years follow up

**Fig. 6:**
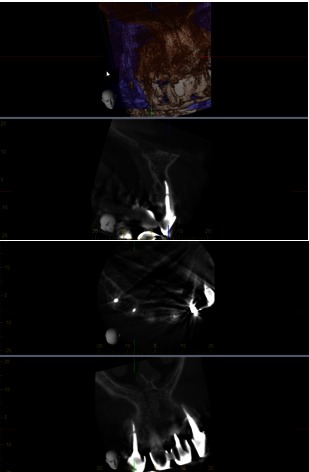
**Fig. 6**CBCT 5 years follow up

After 5 years, the patient was scanned with a CBCT device, Accuitomo, Morita, Japan, and the sagittal slices revealed a significant decreased lesion, but not completely healed, which might have been a fibrous healing. The radiograph showed a complete healing of the periapical lesion. The tooth was also still asymptomatic and functional. The periapical index (PAI) on the preoperative radiograph was 4, at 12 months follow-up (**Fig. 3**), 2 at 18 months (**Fig. 4**) and 1 at 5 years (**Fig. 5**). The CBCTPAI on the preoperative image was 5 and 2 after 5 years (**Fig. 6**).

Comparing the X-ray with the CBCT, it could be concluded that CBCT had a higher sensitivity to diagnose the periapical status.

## Conclusions

This particular case revealed the efficiency of the nonsurgical, single – visit retreatment, with the preservation of the root length, less traumatic therapeutical technique.

The single visit retreatment is considered efficient even though most of the studies revealed more residual microbes, compared to multiple visits treatment. The advantages of single visit are a lower rate of flare-ups and reinfections between appointments and the minimizing of the costs [**5**,**6**,**7**]. There was no reported difference in the outcome of the treatment. The risk of a fracture between appointments was decreasing. The situations in which a multiple visit treatment is required are highly infected canals, when an antimicrobial dressing is applied, excessive exudate, hemorrhage, or the impossibility to achieve complete chemo-mechanical root canal treatment due to the complex morphology or iatrogenies. Multiple visits are recommended in symptomatic retreatment cases [**8**].

The high success rate of conservative retreatment recommends the orthograde approach as a primary measure. In poorly treated canals, like in our case, the microbial flora is similar to that seen in a previously untreated case, so the single visit can be the first choice of treatment.

Due to the higher sensitivity of CBCT and the more accurate detection of the periapical status, the success rate of the endodontic treatment is lower compared with radiographs. The modern conception states that the size decreasing of the initial lesion, associated with asymptomatic and functional tooth is considered an efficient treatment [**9**,**10**,**11**,**12**].

In conclusion, the conservative single visit retreatment should be the first choice in retreatment, when confronted with poorly filled root canal with a large apical lesion. CBCT produces high-resolution images and provides more detailed information about the bone lesions and the outcome of an endodontic treatment. Compared with PA X-ray, CBCT showed a higher accuracy in detecting periapical lesions, canal anatomy, and root canal fillings.
